# Nanoporous silicon nitride-based membranes of controlled pore size, shape and areal density: Fabrication as well as electrophoretic and molecular filtering characterization

**DOI:** 10.3762/bjnano.9.131

**Published:** 2018-05-09

**Authors:** Axel Seidenstücker, Stefan Beirle, Fabian Enderle, Paul Ziemann, Othmar Marti, Alfred Plettl

**Affiliations:** 1 Institute of Solid State Physics, Ulm University, Albert-Einstein-Allee 11, 89069 Ulm, Germany; 2 Institute for Applied Materials, KIT, Hermann-von-Helmholtz-Platz 1, 76344 Eggenstein-Leopoldshafen, Germany; 3 Institute of Experimental Physics, Ulm University, Albert-Einstein-Allee 11, 89069 Ulm, Germany

**Keywords:** ion transport, micellar technique, molecular filtration, nanopores, solid-state membrane

## Abstract

A new route will be presented for an all-parallel fabrication of highly flexible, freestanding membranes with well-defined porosity. This fabrication is based on arrays of well-defined Au nanoparticles (NPs) exhibiting a high degree of hexagonal order as obtained in a first step by a proven micellar approach. These NP arrays serve as masks in a second reactive ion etching (RIE) step optimized for etching Si and some important Si compounds (silicon oxide, silicon nitride) on the nanoscale. Application to commercially available silicon nitride membranes of well-defined thickness, delivers a diaphragm with millions of nanopores of intended and controlled size, shape, and areal density with narrow distributions of these parameters. Electrophoretic transport measurements indicated a very low flow resistance of these porous membranes in ionic solutions as expected theoretically. Size-selective separation of protein molecules was demonstrated by real-time fluorescence microscopy.

## Introduction

Various types of excellent freestanding nanoporous membranes for applications such as charge- and size-based separation of molecules have been reported during the last decade. These membranes exhibit very small thickness and large pore numbers enabling ultralow flow resistance while ensuring good mechanical stability together with chemical inertness. In general, thin single layers made from pure materials such as polymers, silicon, and silicon nitride are used. Recently, ultrathin graphene membranes became very promising for various applications including sequencing. However, the controlled generation of nanometer-size pores is limited to the sequential fabrication of pores [1–3]. But sub-nanometer pores in atomically thin graphene membranes, zeolites, two dimensional polymers, or metal-organic frameworks for the selective filtering of ions and gases can now be prepared with parallel techniques [4–9]. In most cases, the reported production techniques offer specific possibilities for controlling membrane parameters such as thickness, porosity, pore shape and size, or they provide the chance for chemically functionalizing inner surfaces of individual pores. Still another approach came from diverse ion-track etching techniques with a limitation in porosity but several ways to form pore shapes [10–12]. Alternatively, thin porous nanocrystalline silicon (pnc-Si) membranes have been suggested with pores formed in a nc-silicon film sandwiched between nanometer-thick silicon dioxide layers during rapid thermal annealing [13]. This fabrication offers the advantage of full compatibility to semiconductor fabrication techniques. While pore distance and pore size can be controlled within acceptable limits, the generated pores are random in position and the membrane material is fixed [14–18]. To avoid the latter restriction, the well-known preparation techniques of solid-state single pores (by focused ion or electron beams or recently by dielectric breakdown) are superior [10,19–20]. However, their sequential approach to generate arrays of highly defined nanopores restricts them to small numbers of pores or specific arrangements, hindering high throughput [21–22]. With this aspect in mind, parallel fabrication techniques, based on self-assembly of polymeric components, become essential in combination with appropriate etching procedures. For this purpose monolayers of non-close-packed colloids, and diblock or triblock copolymers were used [23–27].

For the present work with targeted pore sizes of some nanometers up to 50 nm in about 50 nm thick membranes, the sought control of pore shape with smooth inner pore surfaces, and, most notably, narrow size distributions of the parameters, a more stable and more perfectly shaped etching mask is needed. Therefore, a mask formed by well-ordered metallic nanoparticles (NPs) was preferred. The chosen etching process is a low-power single-step reactive ion etching (RIE) process with CF_4_/CHF_3_ gas flow and adjustable sample temperature [28–29]. Spherical Au NPs with controlled size and inter-particle distance are fabricated by a well-proven micellar technique [30–35]. These particles should also be applicable as a mask in NP-assisted plasma etching of conical pores in thicker membranes (some 100 nm) from various semiconductor materials [36].

The performance of the nanoporous membrane is studied by electrophoretic measurements. In addition, a comparison with a simple theory that allows simulating the microfluidic setup with inserted membranes of some million pores as well as FIB-drilled single pores is carried out. Subsequently, molecular filtering as a direct application of our nanoporous membranes is demonstrated: Successful size-selective separation of dye molecules and labeled proteins is observed by real-time fluorescence microscopy.

## Results and Discussion

### Fabrication of nanoporous membranes

**Figure 1:**
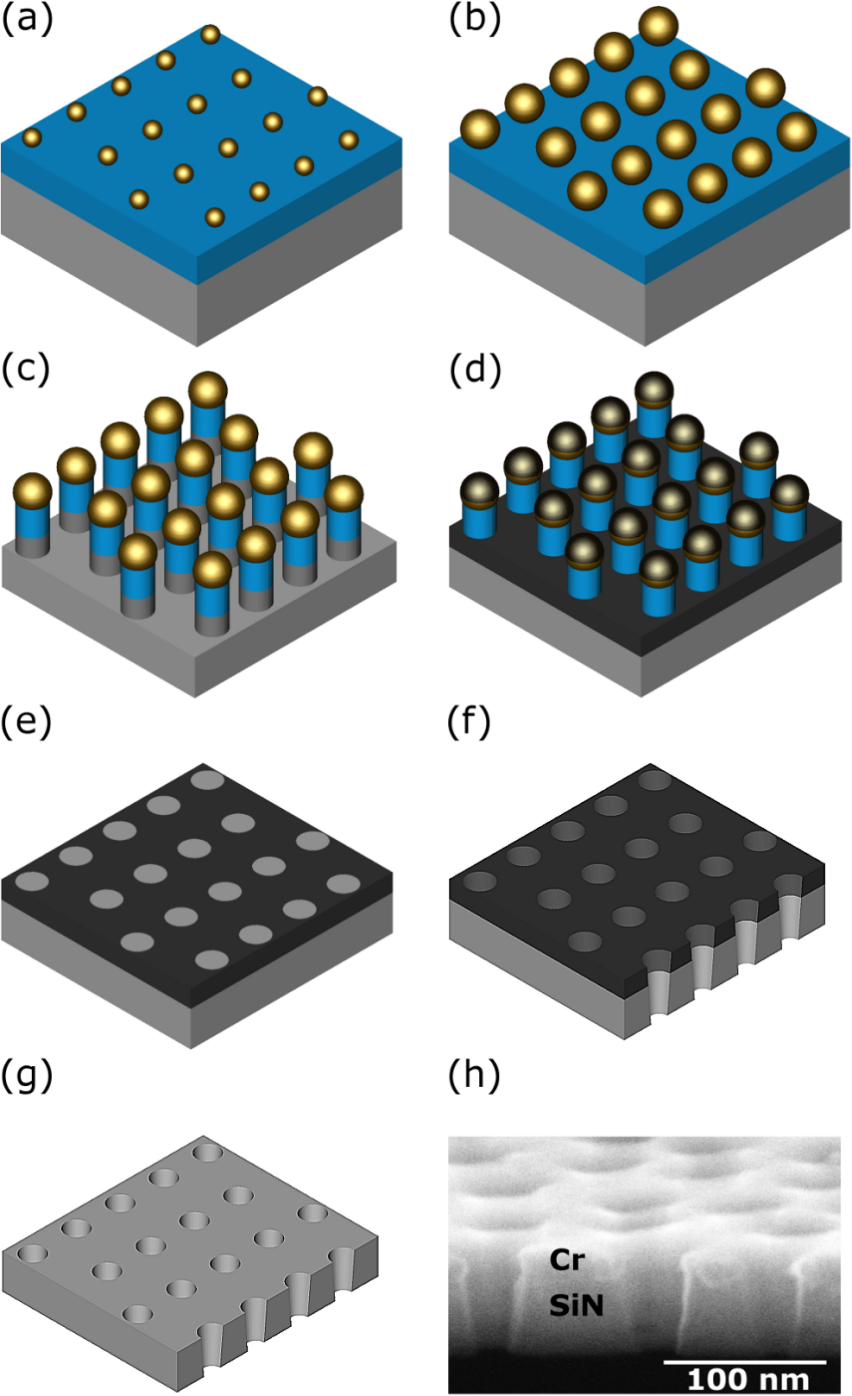
Fabrication scheme for nanoporous membranes: (a) The commercially available silicon nitride membrane (grey) is coated with a sacrificial layer of silicon oxide (blue) on top of which a quasi-hexagonally ordered array of Au NPs (yellow) is deposited by self-organization of Au-loaded diblock copolymer micelles followed by plasma treatments. (b) Applying a photochemical growth process the Au NP size can be enlarged in a controlled way. (c) The resulting NP mask is exploited to etch nanopillars by RIE, (d) a Cr layer (black) is evaporated, (e) and the pillars are removed by grazing incidence sputtering with Ar ions. (f) Subsequent RIE results in nanopores. (g) Optional removal of the residual Cr layer with a liquid etchant. (h) Experiment: SEM micrograph of the cross-section of membrane A (cf. text) with nanopores.

In the following, a description of the various fabrication steps for nanoporous solid-state membranes is given, and their respective additional capabilities are addressed. The all-parallel nanoengineering process starts with a commercial (silicon-rich) silicon nitride (SN) membrane, typically with a size of 500 µm × 500 µm and a thickness of 75 nm supported by a 5 mm × 10 mm Si carrier with a chemically etched pyramidal opening at the back side (Silson Ltd, Northampton, England). The process scheme shown in Figure 1 illustrates the steps necessary to obtain a nanoporous membrane. Experimental details are summarized in Table S1 in Supporting Information File 1.

First, in Figure 1a an approximately 55 nm thick sacrificial layer of silicon oxide is deposited on top of the SiN membrane by electron beam physical vapor deposition (EBPVD). After that, Au NPs (typical diameter ca. 12 nm) are deposited on the silicon oxide layer with a quasi-hexagonal order by self-organization. This is realized by dip-coating from a solution of Au salt-loaded PS–P2VP diblock copolymer micelles directly on the upper membrane face (coating is done without preceding deposition of appropriate polymer brushes [31]). During this process, precise control of the pull-out velocity and the suppression of any vibrations are essential and determine the quality of the homogeneous areal density of the NPs, and thus, the related density of the pores later on. After drying of the micellar film, the removal of the polymers, the reduction of the Au ions, and the formation of spherical Au NPs are obtained by exposure to hydrogen plasma [32]. Subsequently, the particles will serve as an etching mask during the RIE process. Hereafter, examples with 100 nm distance between the centers of adjacent NPs will be presented. The size distribution of the NPs (taken from high resolution SEM top view figures) can be well described by Gaussian distributions. However, occasionally the micellar technique leads to a local aggregation of bigger particles: By employing polymers of high quality and optimized process guidance, such NPs become extremely rare. Additionally, the resulting few larger pores can be sealed by an appropriate e-beam-assisted repair process as demonstrated in Supporting Information File 1. In principle, the size of the NPs can be varied by the choice of specific parameters of the micellar technique [37]. But in practice the size is limited to approximately 10 nm. Therefore, in a subsequent process the ‘micellar’ particles will be enlarged, see Figure 1b. Electroless growth techniques are well-known mostly for particles in solution, but also work for particles previously deposited on substrates [38–39]. However, this approach often does not meet the enhanced requirements on the perfection of the spherical shape and the narrowness of the size distribution of metallic NPs. Hence, we prefer a selective photochemical growth technique of an additional Au shell of homogeneous thickness on top of the micelle-grown Au NPs [35,40]. Up to a diameter of about 30 nm this process can be done in a single step. The spherical shape can be improved by additional annealing [35].

These Au NPs are then applied as an etching mask in a RIE process that was optimized to remove Si and Si compounds (oxide or nitride, amorphous, nanocrystalline or crystalline) anisotropically on the nanoscale using a commercial setup (OXFORD PlasmaLab 80 Plus ICP65, England), compare Figure 1c in [41]. To generate high-aspect-ratio nanopillars, in the first step the sacrificial SiO*_x_* layer is completely removed together with an additional small fraction of the SiN layer. This procedure allows for the inversion of the original etching mask by conversion of nanopillars into a hole-etching mask. For that purpose a Cr layer is deposited in shadow technique by thermal evaporation at room temperature (RT), shown schematically in Figure 1d. The Cr film thickness is determined by the etch selectivity of mask and membrane materials. All the Cr caps on top of the pillars must be clearly separated from the Cr layer on the SiN bottom for the chosen aspect ratio of the pillars. The second part of the mask inversion process (Figure 1e) applies a low current density Ar^+^ ion sputter process under grazing incidence. It results in a Cr pore etching mask with well distributed, smooth, free SiN disk surfaces with diameters (defining the final upper hole diameter) slightly increased in comparison to the size of the Au NPs. This increase is due to the fact that the nanopillars etched in SiO*_x_* are slightly conical. Thus, the electrochemical growth step for the NPs could be substituted or shortened by adjusting the thickness of the SiO*_x_* sacrificial layer. For smaller pore diameters, this enlargement can be minimized by using amorphous Si or SiN as a sacrificial layer. The size distributions of the opened SiN disks are similar to those of the Au NPs.

The membranes are etched with a commercial RIE etcher at RT (cf. Figure 1f, for further details see Supporting Information File 1). The cross section of a nanopore of comparable length in a much thicker homogeneous material such as Si can be approximately described as an ellipse with its main axis perpendicular to the surface [28]. In the present case, the finite thickness of the membrane offers new shaping opportunities by choice of the etching time: One approach is to etch through the complete membrane in a single step till the sought upper or lower pore diameters are reached. For the related pore a conical profile is obtained with well-defined opening angle. In general, the resulting geometry of a pore depends in a complex way on the initial diameter of the mask, the membrane thickness and the material composition as well as the etching time. The SEM image given in Figure 1h shows the cross section of typical pores in a 50 nm thick SiN membrane. To enhance the image contrast, the Cr layer was left on the membrane. Another possibility is to stop the etching process just before the breakthrough occurs, to turn the sample and to start homogeneously thinning the membrane from the back side till the desired pore diameter is reached. This option should be preferred for the generation small pore diameters of some nanometers on the back side, or for membrane thicknesses above 100 nm. It is worth mentioning that opening angles can be enlarged by lowering the sample temperature to about −100 °C during RIE. It also should be noted that the accuracy of the total process depends on the temporal constancy of the etching process. Pre-set values of the average pore diameter are typically obtained with a precision of ±2 nm. Optionally, the Cr film can be removed by using commercial Cr etch (Figure 1g) and finally wet cleaning with pure water in a multistage procedure.

To demonstrate the versatility and power of the described preparation procedure, in the present work the fabrication of 4·10^7^ nanopores was achieved with pore diameters as small as 14 nm on the back side of 500 × 500 µm^2^ membranes corresponding to an areal density of approximately 100 µm^−2^. Area fractions (pore area/total membrane area) on the top and on the bottom faces in the range of 3–15% and 2–5% were obtained, respectively.

**Figure 2:**
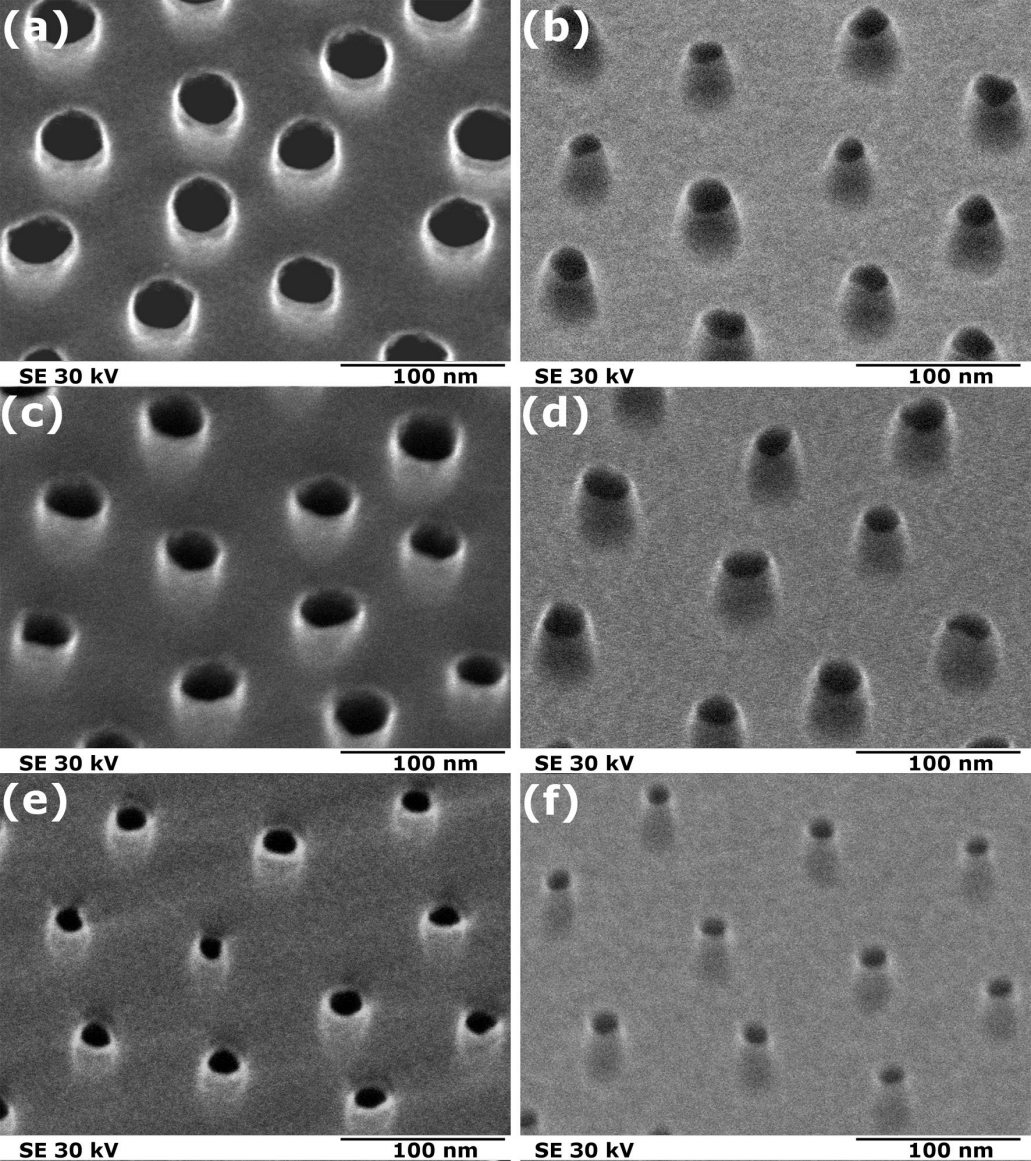
HRSEM micrographs of nanoporous silicon nitride membranes with Cr top layer. Shown are three membranes (A, B, and C) with different average diameters. The left column (a, c, e) presents the top side and the right column (b, d, f) the corresponding bottom side (scale bars: 100 nm). The resulting diameter data are summarized in Table 1.

Three different types of porous membranes were fabricated (Figure 2). The respective preparation parameters are given in Supporting Information File 1 (Table S1). Membranes A, B, and C were patterned with conically shaped nanopores. This nanopore shape was deduced from SEM micrographs of the top and bottom faces of the membranes. The respective diameters are given in Table 1. As a result, the three experimental examples prove the applicability of the proposed parallel processing of membranes with conical nanopores spanning a range of bottom diameters from 22.1 nm (membrane A) down to 13.6 nm (membrane C) each with a narrow size distribution.

**Table 1:** Pore sizes from top view HRSEM images of the analyzed membranes A, B, and C.

membrane	pore diameter (nm)	
	top side	bottom side

A	37.0 ± 4.0	22.1 ± 4.0
B	31.1 ± 2.9	26.2 ± 2.8
C	19.1 ± 1.2	13.6 ± 1.5

**Figure 3:**
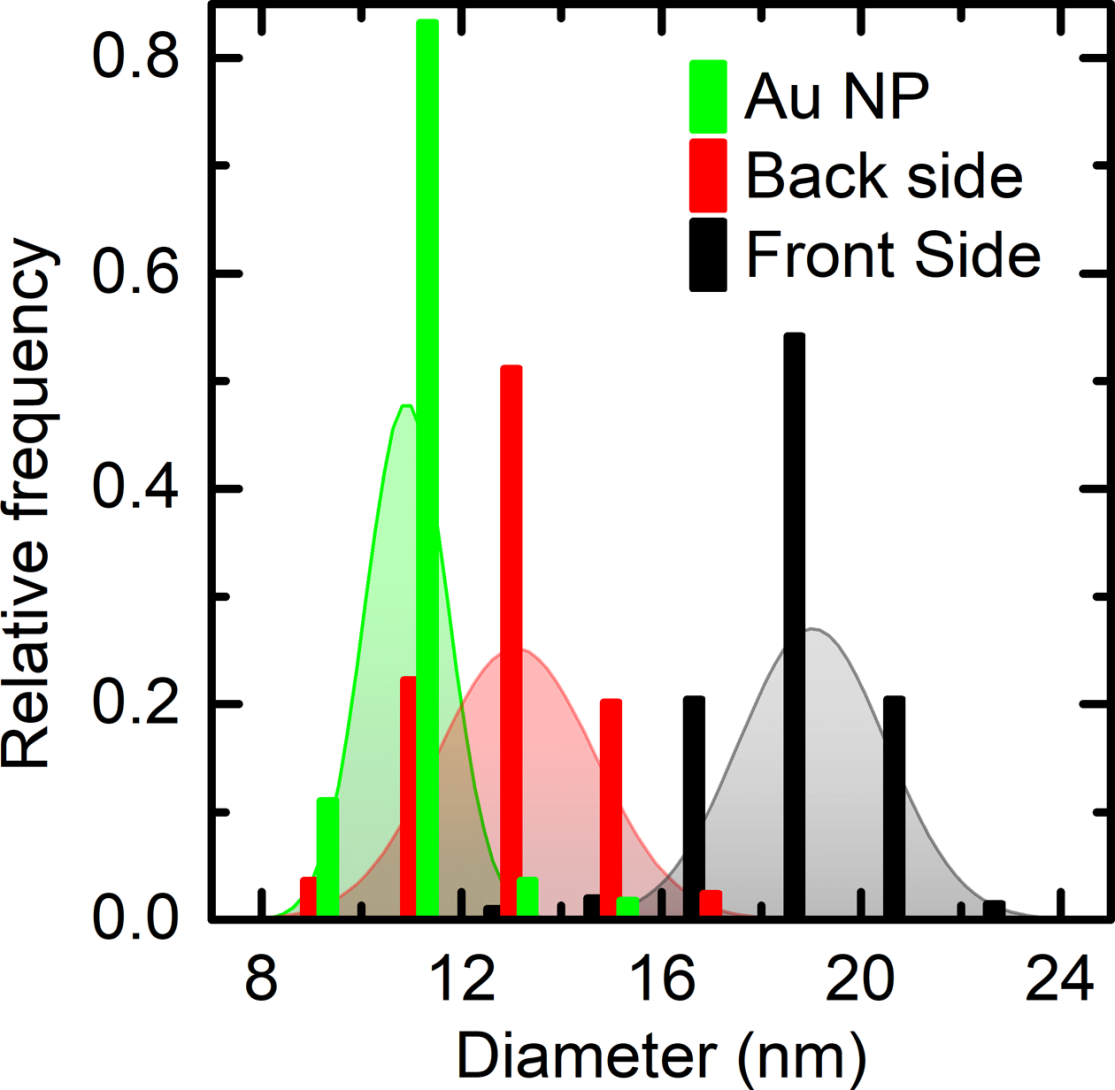
Diameter distributions for the Au NP etch mask (green) and the resulting nanopores of membrane C on the front side (black) and on the back side (red). Vertical bars represent experimental data to which the shaded Gaussians were fitted.

Figure 3 shows the fitted normal distributions of the pore diameters on front and back side of the membrane with the smallest pores (membrane C). For comparison, the corresponding diameter distribution of the Au NPs used as etch mask is added. The fitted distributions were derived from datasets consisting of more than 190 nanopores and more than 100 Au NPs. For each dataset normality tests (Kolmogorov–Smirnov) were applied and could not be rejected at a significance level of 0.01. The narrow size distribution of the NPs at process start of 10.9 ± 0.8 nm results in uniform pore diameters at the top and bottom face of 19.1 ± 1.2 nm and 13.6 ± 1.5 nm, respectively.

### Electrophoretic characterization

**Figure 4:**
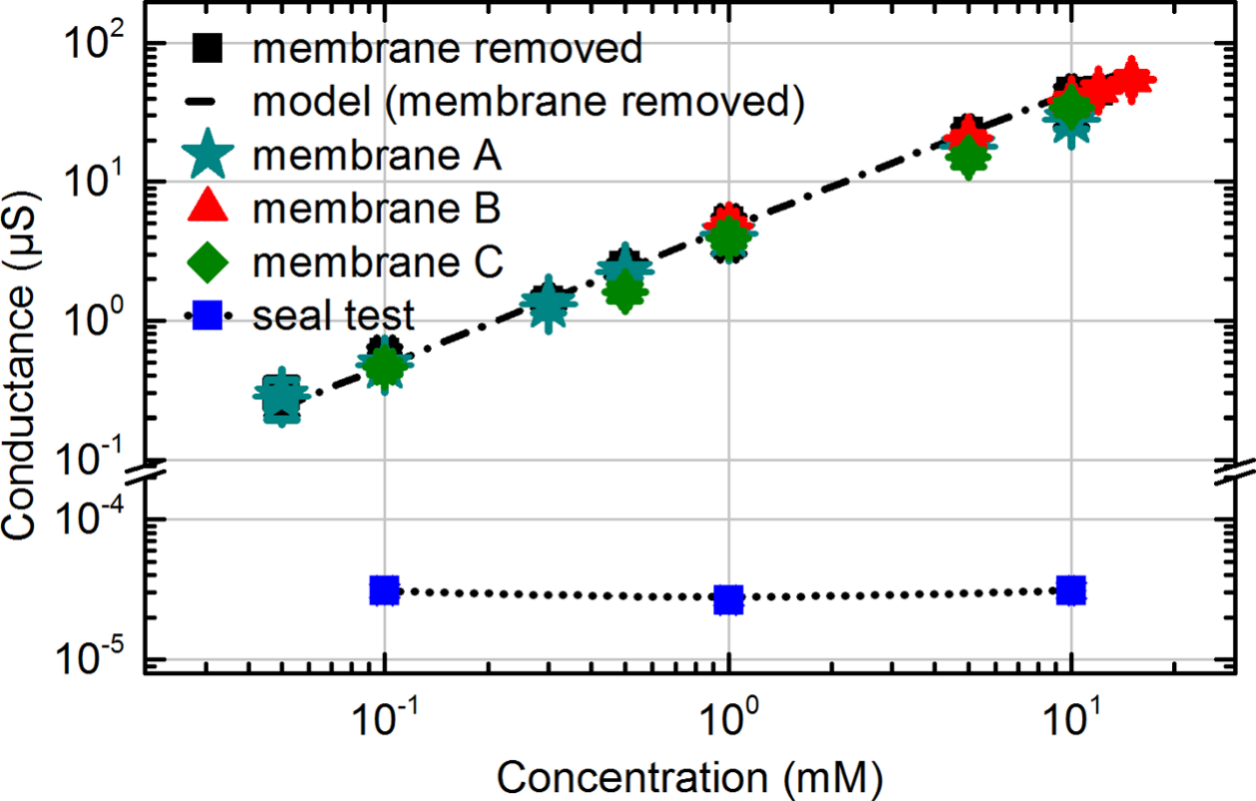
Ionic transport measured through the three membranes A, B and C for KCl electrolyte concentrations in the range of 5∙10^−2^ to 12 mM. Additionally, the measured conductance of the setup without membrane and the modeled one (calculated by FEM) are shown. A seal test with a non-porous membrane reveals the contribution of leakage currents.

After the fabrication of nanometer-sized pores the permeability of the resulting membranes was tested. For this purpose, membranes with an array of millions of nanopores were characterized first by ionic transport measurements. The tests were performed on the three types, A, B and C, of membranes introduced above. The membranes were mounted between a cis- and trans-chamber made of Teflon, each equipped with an Ag/AgCl electrode (Supporting Information File 1, details in the text and Figure S1). Subsequently, the chambers were filled with a KCl electrolyte and the conductance was determined by applying dc voltages to the Ag/AgCl electrodes and automated current measurements. The results of the according experiments on the membranes A, B, and C are shown in Figure 4. Measurements of the fabricated membranes always revealed high ionic conductances. Documented here are the examples A, B, and C in comparison to a membrane without any nanopores (“seal test”). For each of the membranes a conductance was obtained comparable to the measured conductance of the setup with removed membrane, created by completely breaking out the freestanding thin silicon nitride layer from its carrier. Thus, the contribution of the nanopores to the total flow resistance in the test setup is found to be very small. To determine the contribution of leakage to the observed conductance values, a membrane without nanopores was investigated. Leakage currents were always three to four magnitudes smaller than the currents found for membranes with nanopores and will be neglected.

To support the measurements, the entire microfluidic setup with removed membrane was modeled through the finite element method (FEM) using COMSOL Multiphysics (Comsol Multiphysics GmbH, Göttingen, Germany) (see Supporting Information File 1 for further details). Experiment and model fit very well for all geometries chosen. The remarkably high conductance of the porous membranes can be explained by modeling the membrane as a parallel connection of *N* nanopores (experimentally: *N* ≈ 10^7^) leading to a total resistance of:

[1]
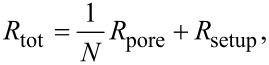


where *R*_pore_ is the resistance of a single nanopore. Its value was successfully approximated by neglecting the influence of surface charges on the pore wall and assuming a homogenous resistivity of the electrolyte; for further details see Supporting Information File 1. Note the following in this context: The applied RIE processes involving CHF_3_/CF_4_ plasmas have a propensity for the formation of Teflon-like CF layers which, by influencing the wettability of the resulting membranes, may deteriorate their permeability. It turned out, however, that fluorocarbon surface contaminations could be removed by annealing in ultra-high vacuum (10^−8^ mbar) at 500 °C for 120 min (details are given in Supporting Information File 1).

For membrane C with the smallest pores the calculated total contribution of the pores *R*_pore_·*N*^−1^ sums up to 1 kΩ (for 1 mM KCl). This resistance value is 200 times smaller than the resistance *R*_setup_ of the complete microfluidic setup without membrane. Therefore, differences of the resistances of the membranes A, B and C due to their varying pore diameters are expected to be smaller than the measurement uncertainties. In accordance with these considerations, the ion transport measurements show a very high overall permeability of the nanopores. As a consequence, a small fraction of possibly blocked pores could not be resolved by the presented measurements of membranes with millions of pores.

### Permselectivity studies by fluorescence microscopy

**Figure 5:**
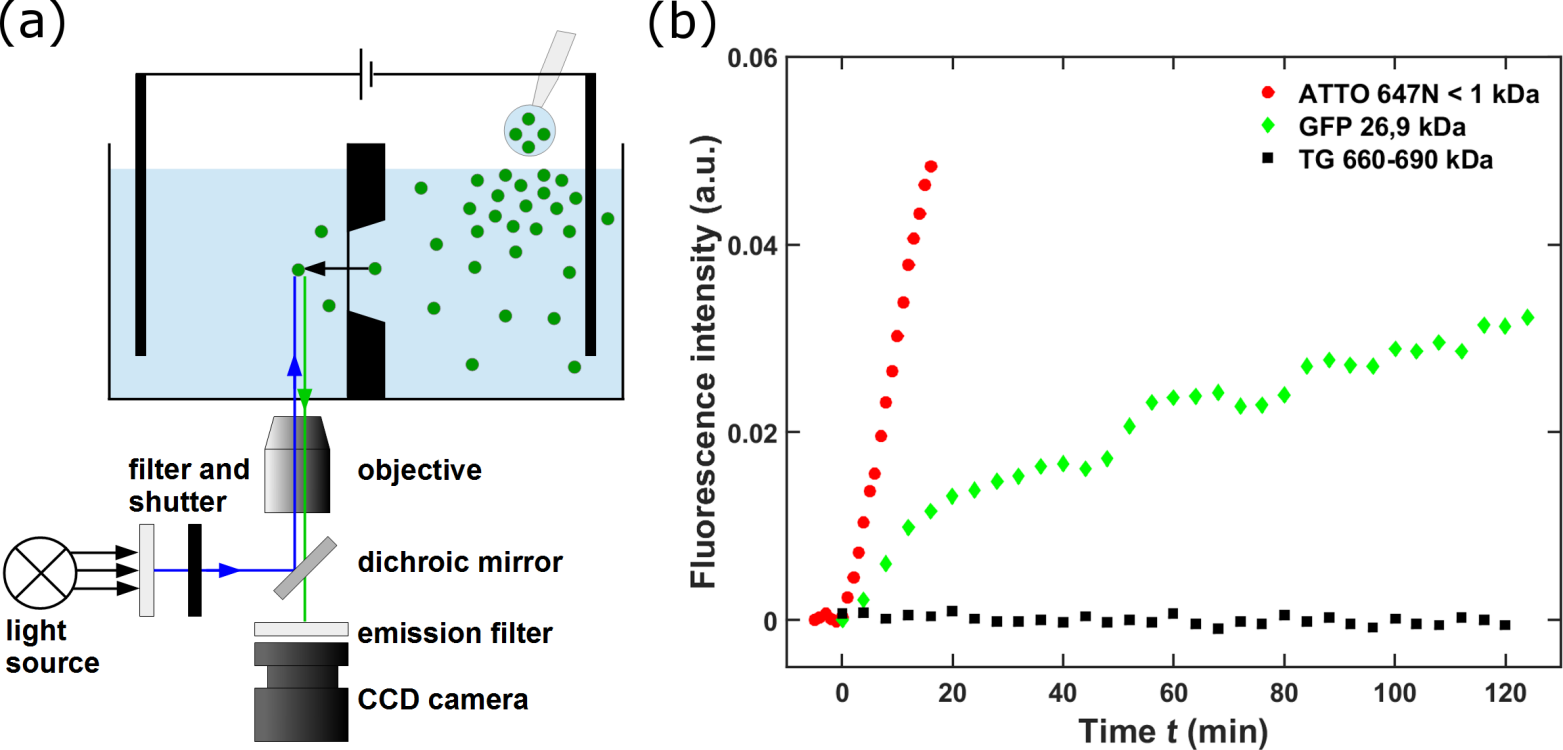
Molecule transport through a porous membrane of type C measured by fluorescent microscopy. (a) Experimental setup, schematically. First a mixture of green GFP and ATTO-labeled TG was applied to the cis fluid reservoir. (b) Emitted intensities of translocated molecules can only be observed for GFP, the larger and heavier TG molecules remain completely in the supply reservoir. After 120 min, the dye molecule ATTO is added and observed instantly behind the membrane.

The main potential of nanoporous membranes will be the sieving of macromolecules in aqueous solution, especially for medical and biological use and even on an industrial scale [15,42–43]. Selectivity and molecular cut-off depend strongly on pore size and the narrowness of its distribution. Studies of the molecular filtering properties were conducted by real-time fluorescence microscopy. Thus, the diffusion of fluorescent molecules or fluorescent-labeled macromolecules could be observed time-resolved. For this purpose a second, optically transparent measurement cell was made from PDMS (polydimethylsiloxane). The fluid channels, each being 11 mm in length, 2 mm in width, and 3 mm in height, are mounted in cis (right side) and trans (left side) configuration to the membrane. Again appropriate electrodes for electrophoretic experiments or for the provision of an external static electrical field (creating the possibility to switch the diffusion of charged molecules) were placed inside this setup (not applied in the present work). Figure 5a schematically shows a cross section through the experimental arrangement: The inverted microscope images the outlet side of the porous membrane (with a working distance (in air) of 4.5 mm). Convenient lamp and emission filters can be placed within seconds for an alternating detection of molecules with distinct optical features. The electromechanical shutter allows for the optimization of the molecule-specific illumination dose. Typically, with a sampling time between 0.5 and 2 min, the total measurement time was varied from 1 to 4 h. The intensity of the fluorescent emission signal was determined by summing up the 16-bit signals of three different pixel areas on the CCD camera array imaging three detection volumes each being 450 µm apart from the membrane level and about 10 × 10 × 1 µm^3^ in size. A measurement starts by applying a droplet of a protein or protein mixture with a pipette to the cis channel and initiating the data acquisition. More experimental information is given in the Supporting Information File 1.

The results presented in Figure 5b form an important proof of principle for the applicability of the nanoporous solid-state membranes introduced in this work as highly selective molecular filters. Permselectivity tests were performed on an additionally prepared membrane of type C containing 4·10^6^ conical pores with an experimentally determined average top side diameter of 17 ± 1 nm and a corresponding back side value of approximately 12 nm. These pores were uniformly distributed over 215 × 215 µm^2^ with an areal density of 87 µm^−2^. In Figure 5b, two experiments are reported: First the diffusion of a mixture of GFP (green fluorescent protein, mass 26.9 kDa) and TG (thyreoglobulin, mass 660–690 kDa) through the membrane is recorded. The green data points represent the time-dependent fluorescence intensity of GFP molecules that have passed the membrane The larger, ATTO-labeled TG molecules, given by the black data points, are completely blocked; only background signals were detected. After 120 min, a droplet of the red dye ATTO (ATTO 647N, mass 843 Da) was added to exclude a relevant influence of ATTO on the diffusivity of the labeled TG. For illustration, the red ATTO points are added using the same time scale as before, but after re-starting the clock. During all these steps, the electrodes included in Figure 5a were not active.

The ATTO-labeled TG molecules with an unlabeled hydrodynamic diameter of 17.16 nm (Sigma, Saint Louis, MO, USA, product information: thyroglobulin from bovine thyroid) are practically the same size as the top side opening of the conical pores but significantly larger than the pores exit on the back side. This size difference of approximately 5 nm, in the present case, is sufficient for the complete retention of ATTO-labeled TG molecules in the cis reservoir. In contrast, the ATTO molecules with typical hydrodynamic diameters in the range of 1.0–1.6 nm [44] are transmitted practically unhindered as indicated by the strong rise of the intensity. The size of the GFP molecules with hydrodynamic diameters between 5.0 and 5.6 nm [45] is between the previous two. Thus, the observed transmittance through the membrane is expected. However, the reduced intensity rate and the shape of the intensity-versus-time curve indicating intermediate restriction steps confirm the general experience “that diffusion may be greatly restricted through pores, even when the pores are many times larger than the diffusing molecules” [46].

## Conclusion

A sequence of parallel preparation processes for porous nano-engineered SiN-based membranes of high versatility is reported. The most relevant achievement is the control over all relevant parameters such as pore shape and size (ranging between 14 and 50 nm) as well as inter-pore distance. By electrophoretic experiments and comparison to theory, the usability of the porous membranes for ionic transport has been proven. Additionally, size-selective filtering has been demonstrated by experimental separation of fluorescent-labeled protein molecules [47]. Although the preparation may be addressed as demanding, the whole processing is factory compatible and stable, enabled by consequent application of parallel processes of unconventional lithography techniques. Inspired by recent reports [48–51], an essential future aim based on the present work is the realization of filters that combine mechanically stable solid-state membranes containing quasi-identical nanopores with the possibility of their additional functionalization by inserting various types of organic moieties. In that case, multifunctional or even switchable filters appear possible.

## Supporting Information

Supporting information features: parameters for the fabrication of the membranes, COMSOL simulations, approximation for the resistance of a membrane with conical nanopores, serial repair mechanism, real-time fluorescence microscopy, and XPS analysis of CHF_3_/CF_4_-etched sample surfaces before and after thermal annealing.

File 1 Additional experimental data.
