# Supplementary material for: Nanoporous silicon nitride-based membranes of controlled pore size, shape and areal density: Fabrication as well as electrophoretic and molecular filtering characterization
Source: Beilstein J Nanotechnol. 2018 May 9;9:1390–8. doi: 10.3762/bjnano.9.131 (PMC6009373; doi:10.3762/bjnano.9.131)
Supplement: File 1 — Additional experimental data. [file Beilstein_J_Nanotechnol-09-1390-s001.pdf]

Supporting Information

for

Nanoporous silicon nitride-based membranes of controlled pore size, shape and areal density: Fabrication as well as electrophoretic and molecular filtering characterization

Axel Seidenstücker*[1], Stefan Beirle[2], Fabian Enderle[1], Paul Ziemann[1], Othmar Marti[3] and Alfred Plettl[1]

Address: [1]Institute of Solid State Physics, Ulm University, Albert-Einstein-Allee 11, 89069 Ulm, Germany; [2]Institute for Applied Materials, KIT, Hermann-von-Helmholtz-Platz 1, 76344 Eggenstein-Leopoldshafen, Germany and [3]Institute of Experimental Physics, Ulm University, Albert-Einstein-Allee 11, 89069 Ulm, Germany

Email: Axel Seidenstücker* - dr.a_seid@gmx.de

* Corresponding author

Additional experimental data

Table S1: Parameters for the fabrication of nanoporous membranes.

Step	Process	Device	Parameters	Materials
a)	- Deposition of a siliconoxide layer	EBPVD (home-made)	55 nm SiO	SiO (99.99%), Umicore
	- Fabrication of gold salt loaded micelles	Magnetic Stirrer	- 1 week for the formation of micelles<br>- 1 week for loading with gold salt	Toluene (VLSI grade), J. T. Baker, Netherlands;<br>PS(1850)-b-P2VP(900), Polymer Source Inc., Canada;<br>Gold(III) chloride hydrate ($HAuCl_4 \cdot H_2O$), Sigma Aldrich, Germany
	- Deposition of micelles	Dip-Coater: M-405.DG,<br>PI, Germany (Translation stage)	Pullout velocity: 5mm·min^{-1}	
	- Reduction of micelles to nanoparticles	TEPla 100-E Plasma Processor,<br>TePla, Germany	H_2 (160W, 90 min, 0.8 mbar)	
b)	- Photochemical growth of the particles	Karl SUSS MJB 3 Mask UV 400,<br>Karl SUSS, Germany	- 5mM $HAuCl_4$/Phtalatester<br>- UV exposure:<br>Membrane A: 8 min exposure time<br>Membrane B: 8 min exposure time<br>Membrane C: 0 min exposure time<br>- acetone and isopropyl bath à 3 min	Phthalatester mixture (product number: 1160), Cargille Labs, USA<br>Acetone and Isopropyl, BASF (VLSI grade)
	- Annealing of the particles	RTP-1200-100,<br>UniTemp GmbH, Germany	800 °C, 45 min (Membrane A and B)	
c)	- Etching of pillars by RIE	Oxford PlasmaLab 80 Plus ICP65,<br>Oxford Instruments, England	CHF_3 and CF_4 plasma<br>(20:2 sccm, 10 mTorr, 25°C,<br>96V bias, 10 min) equals 70 nm pillars	
d)	- Deposition of a chromium layer	PVD (home made)	15 nm Cr	Cr (99.6%), Balzers
e)	- Sputtering of the pillars	IQP 10/63, Specs, Germany	Ar-Ions, 4,5 keV, 84° incidence angle, current density: 15.6 µA·cm^{-2}, 45-60 min sputter time	
f)	- Etching of nanopores by RIE	Oxford PlasmaLab 80 Plus ICP65,<br>Oxford Instruments, England	CHF_3 and CF_4 (20:2 sccm, 10 mTorr)<br><br>Membrane A: -30 V, -100°C, 30 min<br>Membrane B: -30 V, -100°C, 30 min<br>Membrane C: -30 V, -100°C, 40 min	
g)	- Removal of the chromium layer (optional)			Etch No 1, Technic France (Sotrachem), France
h)	- Ion transport measurements	EPC 08, Heka, Germany		

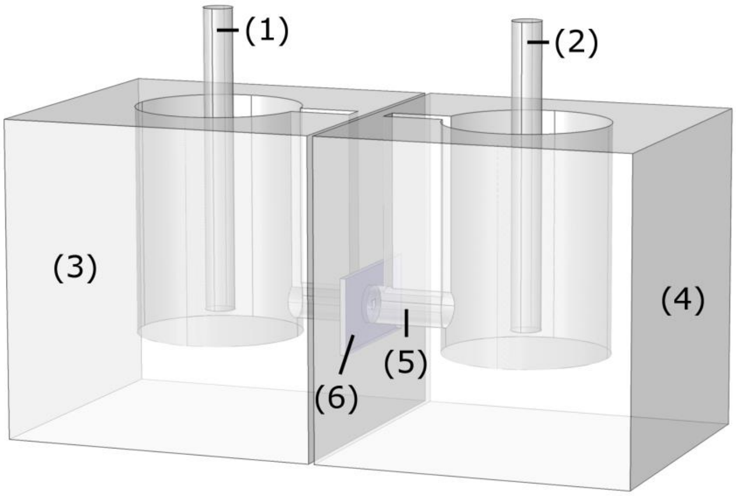

Figure S1: Scheme of the ion transport measurement setup (designed with COMSOL multiphysics). For ion transport measurements the membranes (6) were mounted in a two-piece chamber (3 and 4) made from Teflon and sealed with Teflon tapes. A microfluidic channel (5) filled with a KCl electrolyte (Sigma Aldrich) connects the membrane with electrodes made from Ag/AgCl (EP08, World Precision Instruments, Germany) (1 and 2). The currents and voltages applied to the electrodes were recorded with a patch clamp amplifier (EPC 08, Heka, Germany).

COMSOL simulation

The resistance of the fluid-channel of the measurement setup (Figure S1) was simulated by finite element methods (FEM) using COMSOL multiphysics. For this, the membrane was removed from the silicon carrier. Compared to the high conductance of the electrolyte, the Teflon chambers as well as the silicon frame can be assumed to be insulating. The potentials on the electrodes were set to 0 and 100 mV, respectively. The electric field was assumed to vanish on all surfaces except at the electrodes and the silicon of the carrier. For the resistance of the fluid in the total chamber an ohmic behavior and a homogenous resistivity of the electrolyte (water) were assumed. The simulations show a major potential drop at the pyramidal Si cavity (Figure S2a). In addition to the potential drop the gradient of the electric current density is depicted by white streamlines showing the ion transport through the opening

in the silicon frame. In comparison, a steady potential drop is expected for a measurement setup without the silicon frame (Figure S2b).

To characterize the microfluidic chamber we introduced a geometrical factor that was defined as the ratio of resistance and specific electrical resistance of the electrolyte. By this we determined a geometrical factor of 19.3 ± 0.1 mm^{-1}. The conductivity of the electrolyte was calculated from the Debye–Hückel–Onsager theory.

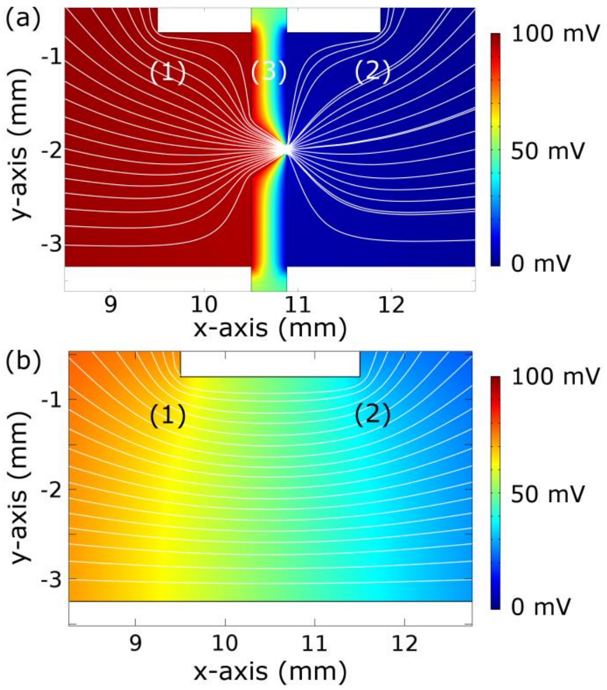

Figure S2: FEM simulations of the measurement setup with mounted silicon frame a) and without frame b). Shown is a detail of the whole measurement setup with the chamber pieces (1) and (2) and the membrane carrier made from Si (3), the electrodes are outside the image shown. The potential is color coded and the gradient of the electric current density is depicted by white streamlines.

Approximation for the resistance of a membrane with conical nanopores

To determine the resistance of a single conical nanopore we are approximating the ion transport by neglecting the influence of surface charges on the pore wall and assuming a homogenous resistivity in the electrolyte. For the resistance of a conical pore we obtain:

$$R_{pore} = \frac{4 \cdot \rho \cdot h}{\pi \cdot a \cdot b}, \tag{S1}$$

with a and b being the diameters of the truncated cone, h the height, and ρ the resistivity of the electrolyte.

The membrane was modeled as a parallel connection of 10^7 nanopores. For the resistance of the total setup follows:

$$R_{tot} = \frac{1}{N} R_{pore} + R_{setup} \tag{S2}$$

with N being the number of pores. Access resistances at the pore orifices were neglected in this approximation since they would only have a minor contribution to the total resistance due to parallel connection.

By measuring the conductance of the electrolyte of a silicon frame with removed membrane we obtained R_{setup}. For the membrane C with the smallest pores the calculated total contribution of the pores $1/N \cdot R_{\text{pore}}$ sums up to 1 kΩ (1 mM KCl). This resistance value is 200 times smaller than the resistance R_{setup} of the complete microfluidic setup without membrane. Therefore, the difference of the membrane resistances due to the varying pore diameters is expected to be smaller than the measurement uncertainties.

Serial repair mechanism

Focused electron beam induced deposition of hydrocarbons (FEBIDH) was applied as a serial repair mechanism to restore membranes showing leakage and tested for tightness. Therefore,

a leak was intentionally formed by drilling a single pore in a 75 nm thick silicon nitride membrane by focused ion beam (FIB) (Helios Nanolab 600, FEI, USA). The pore had a diameter of 162 nm on the top side and 140 nm on the bottom side.

We conducted ion transport measurements the same way as described for the nanoporous membranes at a KCl concentration of 100 mM (Figure S3, open pore). The linear characteristics of the pore are within expectations with a resistance of 7.7 ± 1.1 MΩ [1].
The location of the pore on the membrane was determined by SEM (Hitachi S5200, Japan) at a low magnification and subsequently sealed by exposure to the electron beam at a higher magnification. By this means a higher dose was applied to the pore and the deposition of hydrocarbons was limited to its vicinity. The sealing process lasted a few minutes and was observed with the secondary electron detector of the microscope. Very good seal tightness was achieved. With a resistance of 5200 ± 270 MΩ the tightness of the seal is comparable to that of a fresh membrane without pores, showing a resistance of 11720 ± 390 MΩ.

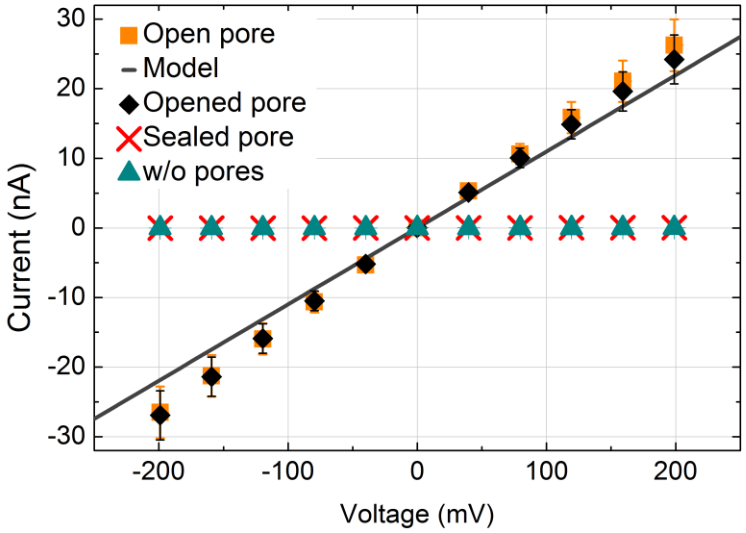

Figure S3: Ion transport measurements of a single nanopore in a silicon nitride membrane. Shown are the *I*–*V* curves for different states of the membrane and a model of a single conical pore. The different states comprise: a fresh membrane without pores (w/o pores), a single freshly drilled pore (open pore), the same pore after sealing (sealed pore), and removal of the seal (opened pore).

To verify the successful sealing of the single pore, the seal, made from hydrocarbon deposits, was removed with oxygen plasma (OXFORD PlasmaLab 80 Plus ICP65, UK). Current measurements showed a resistance of 7.4 ± 1.1 MΩ (opened pore), similar to the pore resistance of the freshly drilled pore.

By taking into account the access resistances at the pore entrances [2]:

$$R_{access} = \frac{\rho}{2}\left(\frac{1}{a} + \frac{1}{b}\right), \qquad \text{(S3)}$$

and Equations S1 and S2 the total resistance was calculated (Figure S3, model).

The experimentally obtained resistances for a single nanopore drilled in a membrane are supported by the good agreement with modeled resistances.

Real-time fluorescence microscopy

The PDMS setup is made by mixing ten parts of Sylgard 184 silicone elastomer and one part of curing agent, degassing for 1 h, giving the material into an aluminum casting mold, and heating at 150 °C for 10 min.

Before each experiment, the hydrophilicity of the areas from PDMS which will be later in contact with water is improved by oxygen plasma (100 W, isotropic, 1 mbar, 5 min). For the same purpose, the wet cleaned membrane is also plasma-treated (hydrogen, 100 W, isotropic, 0.8 mbar, 160 W). Mounting of the device and filling it with PBS (phosphate buffered salt solution) at pH 7.5 is done within 5–10 min. For further reduction of surface tension a 1/50 volume part of 5% polysorbate 20 (TWEEN 20) solution is added. Starting the measurement means adding 0.1–4 µl of a protein solution or 1:1 mixed protein solutions.

In the paper we report on experiments with ATTO, GFP, and ATTO-labeled TG:

ATTO 647N is a red dye, has an atomic weight of 843 Da, excitation maximum λ_{ex} = 644 nm, emission maximum λ_{em} = 669 nm, and a hydrodynamic radius of 0.5–0.8 nm.

GFP (Green Fluorescent Protein) has an atomic weight of 26.9 kDa, λ_{ex} = 395 nm, emission maximum λ_{em} = 475 nm, and a hydrodynamic radius of 2.5–2.8 nm.

TG (Thyreoglobolin) has an atomic weight of 660–690 kDa, and a (unlabeled) hydrodynamic radius of 8.58 nm. It was labeled with ATTO, see above.

The microscope is from Zeiss /Germany: AxioObserver D1 with AxioCam MRC, Objetiv A-Plan 10×/0.25 PH1, and source HXP 120C. Two filter-combinations were used: 450–490 nm/500–550 nm and 625–655 nm/665–715 nm.

XPS analysis of CHF_3/CF_4-etched sample surfaces before and after thermal annealing

After reactive ion etching with CHF_3/CF_4 plasma one has to expect that process-specific, locally varying CF layers will influence the wettability [3-5] and reduce the possibility for complete homogenous chemical functionalization of the inner pore walls. This makes a removal of teflon-like remnants (potentially hydrophobic) a precondition after RIE.

To further characterize the nanoporous membranes an XPS analysis (PHI 5800, Physical Electronics, USA) was conducted. A contamination with a fluorocarbon film originating from reactive ion etching with CHF_3/CF_4 was shown for plane SiN wafer surfaces (Figure S4, curves A and E). Curve A shows the F 1s peak of a SiN surface after etching. Clearly distinguishable are the main peak of the SiF_x compound and its shoulder originating from a CF_x peak. The peaks are in very good agreement with binding energies found in literature [6-10]. As a reference the spectrum of the SiN prior to the plasma was analyzed, where the F 1s signal is extremely weak. This shows that the fluorine present on the samples A to D originates from the CF_4/CHF_3 plasma.

In order to remove the fluorocarbon contamination the surface was annealed in ultra-high vacuum (10^{-8} mbar) at 500 °C for 120 min (curve B). The CF_x peak vanished as desired whereas the SiF_x peak remained unchanged. To remove SiF_x compounds, the surface was

exposed to deionized water for 15 min (C) and 23 h (D) respectively, which resulted in the formation of HF dissolved in water and SiOH [11]. After 15 min the peak was drastically reduced. An increase in the exposure time to 23 h did not succeed in the removal of the whole signal, which might be an indication for fluorine that diffused into the bulk silicon nitride unreachable for water molecules, but within the detection depth of XPS. For a similar system (in Si), a more complete removal of the fluorine peak was achieved at a slightly higher annealing temperature of 550 °C [12].

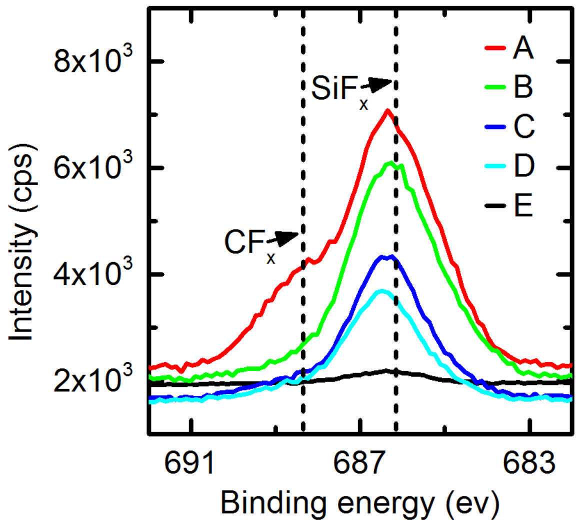

Figure S4: XPS spectra of silicon nitride surfaces. Shown are the F 1s peaks after etching with CHF_3/CF_4 plasma (A), additional thermal treatment at 500 °C in high vacuum for 2 h (B), and subsequent exposure to H_2O for 15 min (C) and 23 h (D). The dashed lines indicate the literature values of binding energies corresponding to the chemically shifted F 1s peaks of CF_x and SiF_x. As a reference a silicon nitride surface was analyzed prior to the etching process (E).

References

1. Smeets, R. M. M.; Keyser, U. F.; Dekker, N. H. *Proc. Natl. Acad. Sci. U. S. A.* **2008,** *105,* 417–421. doi:10.1073/pnas.0705349105
2. Hall, J. E. *J. Gen. Physiol.* **1975,** *66,* 531–532. doi:10.1085/jgp.66.4.531
3. Powell, M. R.; Cleary, L.; Davenport, M.; Shea, K. J.; Siwy, Z. S. *Nat. Nanotechnol.* **2012,** *6,* 798–802. doi:10.1038/nnano.2011.189
4. Smirnov, S.; Vlassiouk, I. V.; Lavrik, N. V. *ACS Nano* **2011,** *5,* 7453–7461. doi:10.1021/nn202392d
5. Smirnov, S.; Vlassiouk, I.; Takmakov, P.; Rios, F. *ACS Nano* **2010,** *4,* 5069–5075. doi:10.1021/nn101080k
6. Bui, L. N.; Thompson, M.; McKeown, N. B.; Romaschin, A. D.; Kalman, P. G. *Analyst* **1993,** *118,* 463–474. doi:10.1039/AN9931800463
7. Nanse, G.; Papirer, E.; Fioux, P.; Moguet, F.; Tressaud, A. *Carbon* **1997,** *35,* 175–194. doi:10.1016/S0008-6223(96)00095-4
8. Beamson, G.; Briggs, D. *J. Chem. Educ.* **1993,** *70,* 25. doi:10.1021/ed070p25
9. Stinespring, C. D.; Freedman, A. *Appl. Phys. Lett.* **1986,** *48,* 718. doi:10.1063/1.96700
10. Chuang, T. J.; Winters, H. F.; Coburn, J. W. *Appl. Surf. Sci. (1977-1985)* **1979,** *2,* 514–531.
11. Ignatov, S. K.; Sennikov, P. G.; Chuprov, L. A.; Razuvaev, A. G. *Russ. Chem. Bull.* **2003,** *52,* 4, 1–9. doi:10.1023/A:1024431805512
12. Jeng, S.-P.; Ma, T.-P.; Canteri, R.; Anderle, M.; Rubloff, G. W. *Appl. Phys. Lett.* **1992,** *61,* 1310–1312. doi:10.1063/1.107575
